# Professional identity and self-rated caregiving performance among work-integrated childcare trainees and early-career practitioners: an indirect association through work engagement

**DOI:** 10.3389/fpsyg.2026.1919887

**Published:** 2026-10-27

**Authors:** Chen Chunhua, He Yuda

**Affiliations:** 1 Zhanjiang Preschool Education College (College of Basic Education, Lingnan Normal University), Zhanjiang, China; 2 Azman Hashim International Business School (AHIBS), University of Technology Malaysia, Kuala Lumpur, Malaysia

**Keywords:** childcare workforce, professional identity, self-rated caregiving performance, structural equation modeling, vocational education, work engagement, work-integrated learning

## Abstract

**Introduction:**

This study examined associations among professional identity, work engagement, and self-rated caregiving performance among childcare trainees and early-career practitioners in China.

**Methods:**

A cross-sectional survey included 318 participants recruited through 11 vocational colleges and 50 childcare institutions in Guangdong Province. Confirmatory factor analysis and structural equation modelling assessed the measurement structure and associations, with bootstrapping and robustness analyses.

**Results:**

The three-factor model showed good fit (CFI = 0.954, RMSEA = 0.039). Professional identity was positively associated with self-rated caregiving performance (total β = 0.694; direct β = 0.465), including an indirect association through work engagement (β = 0.229, bias-corrected 95% CI [0.128, 0.339]). Findings remained consistent across robustness analyses.

**Discussion:**

Professional identity and work engagement were associated with more favourable self-appraisals of caregiving performance. The cross-sectional, single-source design precludes causal inference, and self-rated performance should not be equated with observed caregiving quality.

## 1 Introduction

Early childhood care and education systems depend on the people who deliver care day to day. International evidence has repeatedly shown that structural features of provision, such as qualifications, ratios, and facilities, influence children’s experiences largely through the quality of everyday caregiver–child processes (Melhuish et al., 2015; Slot et al., 2015). Attention has therefore shifted from infrastructure toward workforce characteristics, including how practitioners understand their occupational role and how much energy and involvement they bring to caregiving tasks.

This study concerns a workforce segment that is expanding rapidly in China: individuals entering childcare practice through vocational education pathways. In this article, integrated childcare (托幼一体化) refers to service arrangements in which care and early education for children under three, and in some settings up to age six, are delivered within a single institutional framework rather than being separated into custodial care and preschool education. Such settings include stand-alone childcare centers, childcare classes attached to kindergartens, community-based inclusive childcare points, and health-education integrated institutions. Practitioners in these settings combine health monitoring, safety protection, feeding and hygiene routines, responsive interaction, play and developmental support, and communication with families. This role differs from conventional preschool teaching in its stronger emphasis on physical care, health surveillance, and infant-toddler routines, and in its typically lower ratio of curriculum-based instruction.

Guangdong Province trains large numbers of students in infant and childcare, preschool education, and early education programmes. During internships, employment-linked placements, and early employment, these individuals participate in authentic institutional routines while remaining connected to their colleges. They occupy a boundary position between education and work. Because occupational values and work habits are thought to consolidate during this transition, their professional identity and work engagement are of interest both as educational outcomes and as workforce indicators.

Two provincial reform contexts frame the study without being modeled in it. The first is an integrated vocational training model known in Chinese policy discourse as post–course–competition–certificate–innovation (岗课赛证创), which aligns occupational post requirements, curriculum standards, skills competitions, vocational certification, and innovation-oriented practice so that training corresponds more closely to industry demand. The second is the older adults and young children (一老一小) care agenda, a national policy priority that promotes coordinated, community-based, and digitally supported services for both older people and young children. Neither initiative was measured here; both are described because they shape the placement arrangements through which participants entered practice.

A conceptual clarification is central to this article. Care quality in early childhood settings is conventionally assessed by trained external observers using standardized instruments such as the Classroom Assessment Scoring System (Pianta et al., 2008), the Infant/Toddler Environment Rating Scale (Harms et al., 2017), or the Sustained Shared Thinking and Emotional Well-being scale (Siraj et al., 2015). Self-appraisal cannot substitute for such observation, because practitioners are poorly positioned to evaluate their own interactional quality and because self-ratings are subject to leniency and self-enhancement (Heidemeier and Moser, 2009). The present study therefore does not claim to measure care quality. Its outcome is self-rated caregiving performance: the practitioner’s own appraisal of how well they apply caregiving knowledge, execute caregiving skills, and support children’s development and safety. This is a self-rated job performance construct in the organizational-psychology sense (Viswesvaran et al., 1996), not a process-quality indicator.

Framed this way, the research gap is specific. Work engagement is a well-established correlate of self-rated job performance (Christian et al., 2011; Rich et al., 2010), and professional identity has been linked to engagement in teacher populations (Sun et al., 2022; Yu et al., 2025), including among Chinese preschool teacher trainees (Chen et al., 2025; Zhang et al., 2025) and kindergarten teachers, for whom professional development and commitment predict work performance (Leung and Lam, 2025). What remains unclear is whether these organizational-psychology relationships hold for a workforce that has not yet fully entered employment. Work-integrated trainees differ from established employees in three ways relevant to theory: their occupational membership is provisional rather than settled, their task exposure is short and supervised, and their identity is still forming rather than being defended. Social identity theory implies that identification should be a weaker resource when group membership is provisional, whereas job demands–resources theory implies that engagement effects should be attenuated when task autonomy is low. Testing the identity–engagement–performance sequence in this population therefore examines whether findings from established workforces extend to a boundary population in which both theoretical conditions are weakened. That is the contribution this study attempts.

## 2 Theoretical framework and hypotheses

### 2.1 Self-rated caregiving performance

Quality in early childhood provision is commonly analyzed through the structure–process–outcome distinction (Donabedian, 1988). Structural quality concerns staffing, qualifications, group size, and physical environment. Process quality concerns the interactions, routines, and responsiveness children actually experience. Outcome quality concerns children’s development and wellbeing. European multi-country analyses indicate that structural features shape child outcomes primarily through process quality (Slot et al., 2015), and reviews of the evidence base reach similar conclusions (Melhuish et al., 2015).

The present outcome sits outside this framework. Self-rated caregiving performance is an appraisal made by the practitioner about their own task enactment. Research on self-rated job performance shows that such appraisals correlate only moderately with supervisor and objective criteria and are systematically more lenient (Heidemeier and Moser, 2009; Viswesvaran et al., 1996). They nonetheless carry information: they reflect how practitioners construe their own competence and effort, which is consequential for persistence, help-seeking, and occupational retention. In a training-to-work transition, this self-appraisal is a meaningful proximal variable in its own right, provided it is not read as a proxy for observed quality.

Professional identity should relate to this appraisal. Practitioners who regard childcare as socially valuable, who feel they belong to the occupation, and who intend to remain in it have reason to attend closely to caregiving routines and to interpret those routines as expressions of professional competence rather than as placement obligations.

**H1.** Professional identity is positively associated with self-rated caregiving performance.

### 2.2 Professional identity as a psychological resource

Social identity theory holds that people derive part of their self-concept from membership in valued groups, and that identification shapes attachment, motivation, and behavior (Tajfel and Turner, 1979; Ashforth and Mael, 1989). Occupational identification is a specific case in which the occupation itself supplies the categories through which work is interpreted (Ashforth et al., 2008). In childcare, where work is emotionally demanding, ethically consequential, and relationally intensive, such identification plausibly carries more weight than in less person-centered occupations; studies of Chinese early childhood teachers report associations between professional identity and emotional labor, self-efficacy, and professional development (Xie et al., 2022; Li et al., 2025).

Job demands–resources theory provides the second component. Resources are aspects of the person or the job that support goal attainment, reduce demand costs, and stimulate growth; they predict engagement, which in turn predicts performance-related outcomes (Bakker and Demerouti, 2017). Professional identity qualifies as a personal resource in this framework for a specific reason: it supplies goal-relevant meaning that is stable across the varied and often mundane tasks of caregiving, allowing routine work to be construed as progress toward becoming a competent member of the occupation rather than as a set of discrete obligations.

Combining the two perspectives yields a prediction neither makes alone. Social identity theory explains where the resource comes from but not how it converts into effort; job demands–resources theory explains the conversion but treats resources as given. Their conjunction implies that the identity–performance association should operate substantially, though not exclusively, through engagement, and that this should hold even where occupational membership is provisional — a condition under which social identity theory alone would predict a weak effect.

**H2.** Professional identity is positively associated with work engagement.

### 2.3 Work engagement and self-rated performance

Work engagement is a positive, fulfilling work-related state characterized by vigor, dedication, and absorption (Schaufeli et al., 2002). Meta-analytic evidence links engagement to task performance, contextual performance, and reduced counterproductive behavior (Christian et al., 2011), and engagement predicts performance partly through personal initiative and resource accumulation (Rich et al., 2010; Bakker and Demerouti, 2017). In childcare, vigor supports sustained attention across long routine-based shifts, dedication supports persistence with safety and developmental responsibilities, and absorption supports the observation and interaction on which responsive caregiving depends.

**H3.** Work engagement is positively associated with self-rated caregiving performance.

Taken together, H1 to H3 imply that part of the association between professional identity and self-rated caregiving performance operates through work engagement. Because all variables were measured simultaneously, this is stated as an indirect association within a covariance structure, not as a temporal or causal mechanism.

**H4.** Professional identity has a positive indirect association with self-rated caregiving performance through work engagement.

Figure 1 presents the hypothesized model. It shows the proposed relationships only; estimated coefficients appear in Figure 2 in the Results.

**FIGURE 1:**
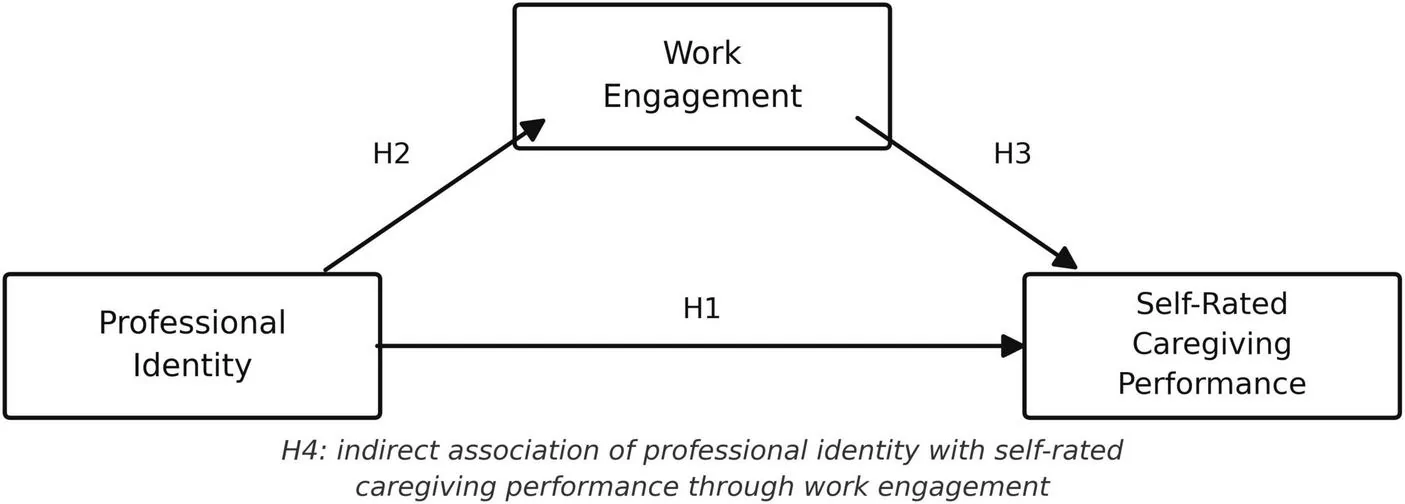
Hypothesized model. The Figure presents proposed relationships without parameter estimates.

**FIGURE 2:**
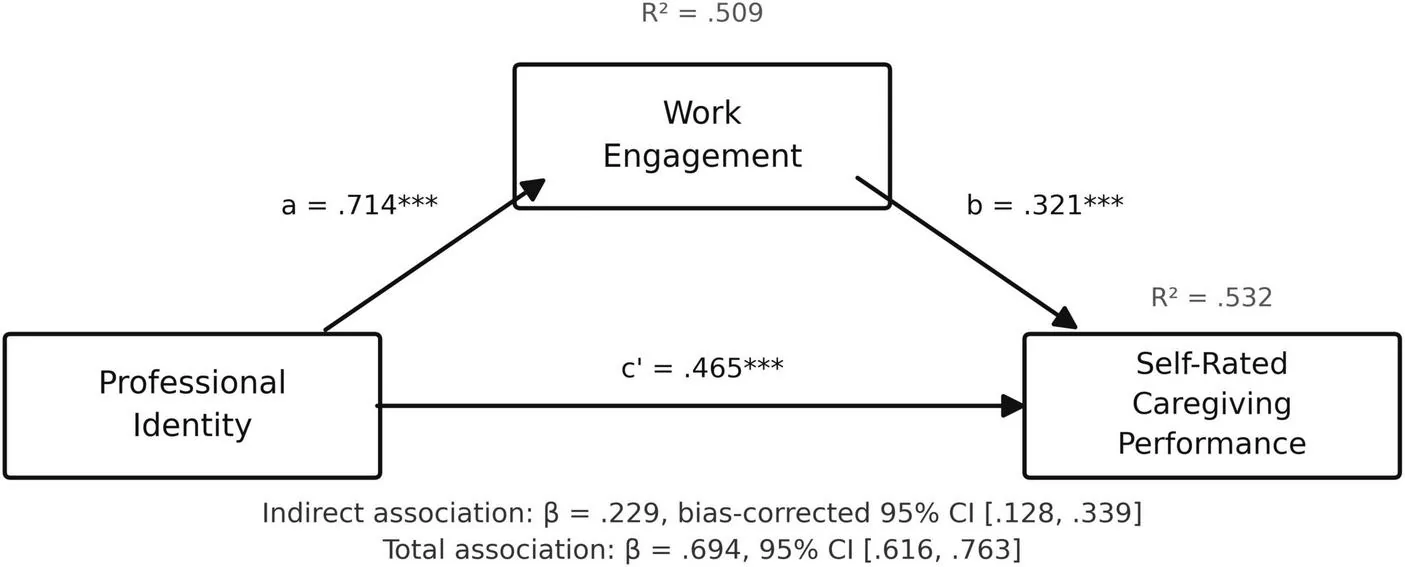
Estimated structural model with standardised coefficients. ***P < 0.001.

## 3 Materials and methods

### 3.1 Design and setting

A cross-sectional online survey design was used. The design permits examination of whether a hypothesized structure is compatible with the observed covariances; it does not establish temporal order and cannot support causal inference. Paths in the model are therefore described as associations throughout.

The study was conducted in Guangdong Province, China. Participants were recruited through 11 vocational colleges in Guangzhou, Shenzhen, Foshan, Dongguan, and Zhanjiang, offering programmes in infant and childcare, preschool education, early education, or related fields. These colleges maintained placement agreements with 50 childcare institutions comprising stand-alone childcare centers, childcare classes attached to kindergartens, community-based inclusive childcare points, and health-education integrated institutions. Children served were predominantly under three years of age.

### 3.2 Participants and recruitment

Questionnaires were distributed between 2 March and 29 April 2026 through institutional and placement communication channels with the assistance of college and practice-site coordinators. Eligibility required current enrolment in, or recent completion of, a childcare-related vocational programme; current participation in an internship, employment-linked placement, or childcare employment; and age of at least 18 years.

Of 352 questionnaires returned, 34 were excluded: 13 for invariant responding across the whole instrument, 10 for missingness exceeding 30% of items, nine for completion times below 150 s, and two as duplicate submissions. The analytic sample comprised 318 responses, a retention rate of 90.3%. The full screening flow appears in Supplementary Table 2. Because the questionnaire was distributed through open placement channels rather than a closed roster, the number of individuals who saw the invitation could not be determined, and a conventional response rate cannot be computed. This is acknowledged as a limitation.

Participation was voluntary and anonymous. No incentives were offered. Coordinators forwarded the survey link but did not receive participant lists, submission records, or any information identifying who responded, so participation or non-participation could not affect academic assessment or placement evaluation. College and institution names were replaced by codes before analysis.

Table 1 reports the sample profile.

**TABLE 1:** Sample profile (*N* = 318).

Variable	Category	*n*	%
Gender	Female	276	86.8
	Male	42	13.2
Age	18–20	179	56.3
	21–23	118	37.1
	24 or above	21	6.6
Major	Infant and childcare	104	32.7
	Preschool education	112	35.2
	Early education	71	22.3
	Other related	31	9.7
Practice status	Internship	198	62.3
	Employment-linked placement	86	27.0
	Employed/early-career practice	34	10.7
Certificate City	Holds childcare certificate	187	58.8
	No certificate	131	41.2
	Guangzhou	116	36.5
	Foshan	87	27.4
	Shenzhen	58	18.2
	Dongguan	29	9.1
	Zhanjiang	28	8.8

Participants were nested in 11 colleges (24–34 participants each) and 50 childcare institutions (2–17 participants each, *M* = 6.4). The female majority is consistent with the demographic composition of childcare training programmes and the childcare workforce in China; generalization to male practitioners is correspondingly limited.

The sample is heterogeneous in practice status, and the label practitioner would overstate the occupational position of the 62.3% currently on internship. Participants are therefore described as work-integrated childcare trainees and early-career practitioners throughout. Descriptive comparisons and robustness analyses across the three practice-status groups are reported in section “4.5 Clustering, practice status, and covariates.”

Information on placement duration, weekly hours, and prior childcare experience was not collected. Because professional identity, engagement, and self-appraisal may all increase with accumulated exposure, this omission limits interpretation and is noted as a limitation.

### 3.3 Sample size and sensitivity

No *a priori* power analysis was conducted; the sample size was determined by the number of eligible respondents obtainable through cooperating institutions within the data-collection window. Adequacy was therefore assessed after the fact by Monte Carlo simulation rather than by deriving a detectable effect from the achieved standard error, which would use the observed result to justify itself.

Following Muthén and Muthén (2002), population values were set to the parameter estimates of the fitted model and complete data were generated from the implied mean vector and covariance matrix. The measurement and structural model was then re-estimated in each replication. Five sample sizes were examined (*N* = 150, 200, 250, 318, 400) with 500 replications each and 1,000 at *N* = 318, giving 3,000 replications in total. Results appear in Supplementary Table 8.

At *N* = 318 every replication converged, and relative parameter bias was below 1.5% for all structural parameters (a = −0.04%, b = +0.90%, c′ = −0.73%, indirect = +0.82%). The empirical standard deviation of the indirect association across replications was 0.0548, which matches the bias-corrected bootstrap standard error of 0.055 obtained from the observed data; these two quantities are derived independently, so their agreement provides a check on the precision reported in section “4.7 Structural model.” Empirical power for the indirect association was 0.990 at *N* = 318 and 0.814 at *N* = 150, so the achieved sample provides comfortable precision for an association of the observed size. We note the corresponding limitation: because population values were taken from the fitted model, the simulation establishes that the model is estimated accurately and precisely at this sample size, not that the sample would suffice to detect a substantially smaller association. Detecting an indirect association half the observed size would require a considerably larger sample.

### 3.4 Measures

All items were administered in Chinese on a five-point Likert scale (1 = strongly disagree to 5 = strongly agree), with higher scores indicating stronger agreement. No items were reverse-scored. Complete Chinese items with English translations, source mapping, and dimensional assignment are provided in Supplementary Tables 1a–c.

Two translation processes must be distinguished. The instruments administered to participants were Chinese; the Chinese items appear in Supplementary Tables 1a–c. English renderings were prepared after data collection for reporting purposes only, using forward translation by one bilingual researcher, review and back-translation of key terms by a second, and reconciliation by discussion. The English versions were never administered and therefore provide no validation evidence for the Chinese instruments.

Content validity was established before data collection. Seven reviewers — four early childhood education scholars, two childcare center directors, and one organizational psychologist — examined the draft items of all three instruments online, rating each for content relevance, clarity of wording, and freedom from ambiguity, and proposing revisions where wording could be misread. The revised instrument set was then piloted with 25 childcare trainees drawn from the same programme population but not included in the main sample. Following expert and pilot feedback, six items were reworded to improve clarity and remove ambiguity (PI2, PI3, WE2, WE5, SRCP8, and SRCP9; marked in Supplementary Tables 1a–c). These revisions affected wording only, leaving item content, dimensional assignment, and scoring direction unchanged. Pilot responses were used solely to assess comprehensibility and appear in no analysis reported here.

This procedure supports the content validity and comprehensibility of the instruments. It does not establish criterion validity: no independent performance indicator was available against which the newly developed self-rated caregiving performance scale could be benchmarked, and this remains a limitation.

#### 3.4.1 Professional identity

Nine items covering professional value identification, occupational belonging, and career commitment. The three-dimensional structure of Wei et al. (2013) provided the primary framework, with item wording informed by Zeng and Liu (2024) and other recent professional-identity research. Teaching referents were replaced with childcare and caregiving referents. The number of items and the scoring direction were not changed. Because wording was drawn from more than one source and adapted to a new occupational context, the instrument is treated as a newly adapted measure rather than an established scale.

#### 3.4.2 Work engagement

Twelve items covering vigor, dedication, and absorption, adapted from the Utrecht Work Engagement Scale (Schaufeli et al., 2002, 2006). For each of the three dimensions, the four statements judged most compatible with childcare practice were selected and adapted, yielding a balanced 12-item version. Adaptation replaced generic work referents with childcare-practice referents and client referents with children and families.

#### 3.4.3 Self-rated caregiving performance

Nine items developed for this study, covering caregiving knowledge application, caregiving skills, and developmental and safety support. Domains were derived from the structure–process–outcome framework (Donabedian, 1988) and the nurturing care framework (WHO, UNICEF, and World Bank Group, 2018), then written as self-appraisal statements about the practitioner’s own task enactment. The scale is new, and the evidence reported here (expert content review, pilot testing for comprehensibility, internal consistency, factor structure, and comparison against merged alternatives) constitutes initial rather than conclusive validation. Item SRCP6 (“I can complete practical care tasks with confidence”) is conceptually adjacent to self-efficacy; it was retained because removing it did not materially alter the factor structure, but its presence is acknowledged when interpreting the construct.

### 3.5 Analytic strategy

Analyses were conducted in Python 3.12 using maximum likelihood estimation. Descriptive statistics, reliability coefficients, and correlations were computed first. The measurement model was then evaluated by confirmatory factor analysis and compared both against merged alternatives, which test whether the three constructs are separable, and against dimensional alternatives, which test whether the sub-dimensions attributed to each instrument are supported. Discriminant validity was assessed by pairwise Fornell–Larcker comparison and by the heterotrait–monotrait ratio with bootstrap confidence intervals. Clustering was examined through intraclass correlations and design effects at college and institution level, through cluster-robust standard errors, and through a cluster bootstrap of the indirect association. The structural model was estimated by full information maximum likelihood, with the indirect association tested by bias-corrected bootstrapping in which each resample was re-estimated by FIML. Covariate robustness was assessed within the latent-variable framework. Analysis code is provided as a standalone file and in the Supplementary material to permit replication in lavaan, Mplus, or AMOS.

Cluster-robust standard errors were computed as the CR1 sandwich estimator, with the finite-sample adjustment G/(G−1) × (n−1)/(n−k), clustering on college; the CR2 bias-reduced estimator was computed alongside it, and inference was referred to a t distribution with G−1 = 10 degrees of freedom rather than to the normal. The implementation was written in Python and reproduces the values returned by sandwich::vcovCL in R. The 50 childcare institutions are nested within the 11 colleges rather than crossed with them: every institution in the dataset is associated with exactly one college. Because adjusting the standard errors of individual paths does not by itself establish that inference for the indirect association reflects the sampling structure, the indirect association was additionally re-estimated in a college-level cluster bootstrap, resampling colleges with replacement.

Missing data were minimal: 79 of 9,540 item responses (0.83%), affecting 56 participants across 32 missingness patterns. Full information maximum likelihood was used, retaining all 318 cases. Listwise deletion (*n* = 262) produced latent correlations differing by no more than 0.02 and identical substantive conclusions.

Distributional assumptions were examined before estimation. Item skewness ranged from −0.86 to 0.14 and excess kurtosis from −0.77 to 0.55, within limits at which maximum likelihood performs adequately with five-point items (Supplementary Table 3). Model fit was assessed using the model χ^2^ with degrees of freedom, CFI, TLI, RMSEA, and SRMR. No post hoc modifications were made: no correlated residuals were freed and no items were removed after inspecting fit.

Common-method concerns were addressed procedurally and statistically, following Podsakoff et al. (2012). Procedurally, participation was anonymous, no incentives were offered, construct blocks were separated and presented in a fixed order with instructional buffers, and respondents were told there were no right answers. Statistically, a common latent factor was added to the measurement model and full-collinearity variance inflation factors were computed. As explained in section “4.6 Common-method variance,” these diagnostics constrain but cannot eliminate common-method explanations.

### 3.6 Ethical considerations

The study was conducted as a sub-study of a multi-site adult research programme entitled Human Resource Development, Educational Innovation, Occupational Wellbeing, Digital Learning and Career Development: A Multi-site Adult Research Programme in China and Overseas Contexts, reviewed and approved by the Scientific Research Ethics Committee of South China Normal University (Approval No. KY2025062302; protocol version 1.0 dated 12 February 2025; approval issued 26 February 2025). The approved research period extends from 1 March 2025 to 30 December 2026, and the approved settings include childcare and early childhood education organizations in China where institutional permission has been obtained. Both the pilot administration and the main data collection, which ran from 2 March to 29 April 2026, fall within the approved period and settings.

The approval covers adult participants only. All participants in the present sub-study were adults aged 18 or above, and eligibility screening excluded anyone below that age. Informed consent was obtained electronically before questionnaire access. No minors or vulnerable participants were recruited, the survey was anonymous and non-interventional, and no personally identifiable information was collected or reported.

## 4 Results

### 4.1 Descriptive statistics and reliability

Construct means fell slightly above the scale midpoint (Table 2), indicating moderately favorable appraisals rather than uniformly high self-ratings. Internal consistency was good for all three scales. Correlations among composite scores were positive and significant.

**TABLE 2:** Descriptive statistics, reliability, and correlations (*N* = 318).

Construct	*M*	SD	α	1	2	3
1. Professional identity	3.38	0.77	0.879	–	–	–
2. Work engagement	3.45	0.77	0.914	0.639**	–	–
3. Self-rated caregiving performance	3.45	0.70	0.873	0.601**	0.575**	–

**P < 0.01. Correlations are based on composite scores. Latent correlations from the measurement model are reported in Table 5.

### 4.2 Measurement model

The three-factor model showed good fit: χ^2^(402) = 595.04, *p* < 0.001, χ^2^/df = 1.48, CFI = 0.954, TLI = 0.950, RMSEA = 0.039, SRMR = 0.040. All standardized loadings were significant at *p* < 0.001, ranging from 0.613 to 0.720 for professional identity, 0.622 to 0.749 for work engagement, and 0.599 to 0.754 for self-rated caregiving performance. Reliability and convergent validity are summarized in Table 3, and complete item-level loadings with standard errors and residual variances appear in Supplementary Tables 1a–c.

**TABLE 3:** Measurement model: reliability and convergent validity.

Construct	Items	Loading range	α	CR	AVE
Professional identity	9	0.613–0.720	0.879	0.880	0.450
Work engagement	12	0.622–0.749	0.914	0.916	0.477
Self-rated caregiving performance	9	0.599–0.754	0.873	0.871	0.431

CR, composite reliability; AVE, average variance extracted. Model fit: χ^2^(402) = 595.04, *p* < 0.001; CFI = 0.954; TLI = 0.950; RMSEA = 0.039; SRMR = 0.040.

Average variance extracted fell below the conventional 0.50 threshold for all three constructs (0.450, 0.477, 0.431). This reflects loadings that are consistently moderate rather than the presence of weak or misspecified items: no loading fell below 0.599, and none was non-significant. Fornell and Larcker (1981) note that convergent validity may still be regarded as adequate when average variance extracted is below 0.50 but composite reliability exceeds 0.60; composite reliabilities here were 0.880, 0.916, and 0.871. The constructs are nonetheless measured with more indicator-specific variance than is ideal, and this is carried into the interpretation of the structural estimates.

### 4.3 Alternative measurement models

Acceptable fit of a hypothesized model does not demonstrate that its factors are empirically separable. The three-factor model was therefore compared against three two-factor models and a single-factor model (Table 4 and Supplementary Table 4 provides the full indices).

**TABLE 4:** Comparison of alternative measurement models (*N* = 318).

Model	χ ^2^	df	CFI	TLI	RMSEA	SRMR	Δχ ^2^	Δ df	Δ CFI
Three-factor (hypothesized)	595.04	402	0.954	0.950	0.039	0.040	–	–	–
Two-factor (PI + SRCP merged)	881.11	404	0.886	0.877	0.061	0.056	286.07	2	−0.068
Two-factor (PI + WE merged)	943.50	404	0.871	0.861	0.065	0.060	348.45	2	−0.083
Two-factor (WE + SRCP merged)	1010.88	404	0.855	0.844	0.069	0.066	415.84	2	−0.099
One-factor	1286.10	405	0.790	0.774	0.083	0.075	691.06	3	−0.164

PI, professional identity; WE, work engagement; SRCP, self-rated caregiving performance. All Δχ^2^ tests *p* < 0.001, evaluated against the three-factor model.

Every merged alternative fitted significantly worse. Merging professional identity with self-rated caregiving performance produced Δχ^2^(2) = 286.07, *p* < 0.001, and ΔCFI = −0.068, far exceeding the −0.01 criterion conventionally used to indicate meaningful deterioration. The single-factor model fitted poorly in absolute terms (CFI = 0.790). These comparisons provide direct evidence that the three constructs are empirically distinguishable and that the associations reported below are not an artifact of a single undifferentiated self-evaluation factor.

These comparisons establish that the three constructs are separable; they do not establish whether the three sub-dimensions attributed to each instrument are supported, which is a distinct question. Each instrument was therefore also tested against its own dimensional structure (Supplementary Table 9). Within each construct, a model with three correlated dimensions fitted significantly better than a single dimension: professional identity, Δχ^2^(3) = 44.18; work engagement, Δχ^2^(3) = 36.60; self-rated caregiving performance, Δχ^2^(3) = 64.63, all *p* < 0.001. A model with all nine dimensions as correlated first-order factors fitted better than the three-factor model, χ^2^(369) = 424.92, CFI = 0.987, RMSEA = 0.022, and a second-order model placing three dimensions under each construct fitted almost as well, χ^2^(384) = 444.77, CFI = 0.986, RMSEA = 0.022. The second-order restriction was not rejected against the unrestricted nine-factor model, Δχ^2^(15) = 19.84, *p* = 0.178, so the three second-order constructs are supported.

The dimensions are therefore empirically identifiable, and the three-factor model used here is a simplification of a structure with more detail in it. We retain the three-factor specification as the primary model because the hypotheses concern the three broad constructs rather than their components, because the second-order test confirms that those constructs are well-defined, and because the structural conclusions do not depend on the choice: estimated from the second-order model, the paths are a = 0.746, b = 0.323, c′ = 0.492, with an indirect association of 0.241 and a total of 0.733, all slightly larger than the first-order estimates but identical in sign and significance. We report the first-order estimates in preference to the larger second-order values, and we note in the limitations that treating each construct as unidimensional understates the measured structure.

### 4.4 Discriminant validity

Discriminant validity evidence was mixed and is reported in full (Table 5 and Supplementary Table 5). The Fornell–Larcker criterion was evaluated pair by pair rather than inferred from the average variance extracted values, and the outcome differs across pairs. For professional identity and work engagement the latent correlation of 0.714 exceeds both square roots (0.671 and 0.691), and for professional identity and self-rated caregiving performance the correlation of 0.694 exceeds both (0.671 and 0.656); neither pair satisfies the criterion. For work engagement and self-rated caregiving performance the correlation of 0.653 falls below both square roots (0.691 and 0.656), so this pair does satisfy it, although by a margin of only 0.003 against the smaller value. A previous version of this manuscript stated that the criterion failed for all three pairs; that statement was inferred from the below-threshold average variance extracted values rather than from the pairwise comparisons and was incorrect, and it is corrected here.

**TABLE 5:** Latent correlations, Fornell–Larcker comparison, and HTMT ratios.

Pair	Latent *r*	√AVE (first)	√AVE (second)	Fornell–Larcker	HTMT	HTMT 95% CI
PI – WE	0.714	0.671	0.691	Not satisfied	0.712	[0.643, 0.774]
PI – SRCP	0.694	0.671	0.656	Not satisfied	0.687	[0.607, 0.759]
WE – SRCP	0.653	0.691	0.656	Satisfied	0.645	[0.560, 0.719]

HTMT confidence intervals from 5,000 bootstrap resamples. Full-collinearity variance inflation factors were 2.41 (PI), 2.28 (WE), and 2.19 (SRCP), all below 3.3.

The heterotrait–monotrait evidence was more favorable. All ratios fell below the 0.85 criterion proposed by Henseler et al. (2015), and all upper confidence bounds (0.774, 0.759, 0.719) also fell below that threshold, indicating that the ratios are reliably below the criterion rather than marginally so. Read together with the model comparisons in Table 4, the evidence indicates that the constructs are separable but share substantial variance. The most defensible reading is that these are distinct constructs measured with imperfect precision through a shared self-report method, and structural estimates involving professional identity and self-rated caregiving performance should be interpreted with this proximity in mind.

### 4.5 Clustering, practice status, and covariates

Participants were nested within 11 colleges and 50 institutions, so independence could not be assumed. Intraclass correlations and design effects were computed at both levels (Table 6), and robustness analyses are summarized in Supplementary Table 6.

**TABLE 6:** Intraclass correlations and design effects.

Construct	College ICC	College DEFF	Institution ICC	Institution DEFF
Professional identity	0.000	1.00	0.000	1.00
Work engagement	0.015	1.41	0.008	1.04
Self-rated caregiving performance	0.034	1.96	0.009	1.05

Colleges: 11 clusters, 24–34 participants each. Institutions: 50 clusters, 2–17 participants each (*M* = 6.4). DEFF, design effect.

Clustering was slight. The largest intraclass correlation was 0.034 for self-rated caregiving performance at college level, yielding a design effect of 1.96 and implying that standard errors could be understated by roughly 40% in the worst case. Institution-level clustering was negligible. Cluster-robust standard errors clustered on college confirmed this: for professional identity predicting self-rated caregiving performance, the conventional standard error of 0.056 became 0.060 under CR1 and 0.060 under CR2; for work engagement, 0.056 became 0.070 and 0.071; and for professional identity predicting work engagement, 0.043 became 0.049 under both. Referred to a t distribution with 10 degrees of freedom, the most conservative of these tests remained significant, *p* = 0.001. A college fixed-effects specification produced closely comparable coefficients (β = 0.397 and β = 0.299).

Because adjusting individual path standard errors does not establish that inference for the indirect association reflects the sampling structure, the indirect association was re-estimated in a college-level cluster bootstrap with 1,000 resamples of the 11 colleges. The cluster-adjusted estimate was β = 0.229, SE = 0.060, bias-corrected 95% CI [0.120, 0.353], against [0.128, 0.339] from the ordinary bootstrap; the standard error rises from 0.055 to 0.060 and the interval is about 10% wider, and it continues to exclude zero. This check is itself limited: with only 11 clusters, both the cluster-robust standard errors and the cluster bootstrap rest on small-sample approximations, and the interval endpoints should be read as indicative rather than exact.

Practice-status groups did not differ significantly on any construct: professional identity, *F*_(2, 315)_ = 0.47, *p* = 0.627, η^2^ = 0.003; work engagement, *F*_(2, 315)_ = 1.05, *p* = 0.350, η^2^ = 0.007; self-rated caregiving performance, *F*_(2, 315)_ = 0.44, *p* = 0.643, η^2^ = 0.003. These are failures to detect differences rather than demonstrations of equivalence. With 34 participants in the employed subgroup, the comparisons have limited power, and differences of a size that would matter substantively cannot be excluded; no equivalence testing was conducted.

Covariate robustness was assessed within the latent-variable framework rather than on composite scores, so that the coefficients are directly comparable in metric with the primary model. A multiple-indicator multiple-cause specification was estimated by full information maximum likelihood on all 318 cases, regressing the three latent variables on twelve covariates: gender, age band, certificate status, major (three indicators), practice status (two indicators), and city (four indicators). The structural paths were essentially unchanged: professional identity to work engagement moved from β = 0.714 to β = 0.728, work engagement to self-rated caregiving performance from β = 0.321 to β = 0.302, and the direct path from β = 0.465 to β = 0.471, giving an indirect association of β = 0.220 against β = 0.229 in the primary model. No coefficient moved by more than 0.02.

An additional check using composite scores in an observed-variable path model is reported in Supplementary Table 6. Coefficients in that metric are systematically smaller than the latent estimates because composite scores are not corrected for measurement error; the comparison of interest there is the change between the composite-score baseline and the composite-score covariate model, not the difference between either of those and the latent estimates. On that basis the paths were also stable, moving from β = 0.395 to β = 0.388, from β = 0.323 to β = 0.308, and from β = 0.639 to β = 0.647. The primary model is reported without covariates. These analyses show that the estimates are insensitive to the specific covariates included; they do not establish independence from background composition in general, since unmeasured characteristics such as placement quality and prior experience were not available.

### 4.6 Common-method variance

Adding an orthogonal common latent factor changed standardized loadings by between 0.006 and 0.043 (*M* = 0.019), below the 0.20 threshold sometimes used as a warning signal, and full-collinearity variance inflation factors ranged from 2.19 to 2.41, below 3.3. These diagnostics indicate that the data are not dominated by a single method factor. They do not, and cannot, demonstrate that common-method variance is absent. All constructs were reported by the same individuals, using the same response format, in a single questionnaire, at one point in time, so the design is susceptible to common-method variance and the associations reported here may be inflated. The design does not by itself establish the magnitude or direction of any such inflation, and the diagnostics reported above do not resolve the question.

### 4.7 Structural model

The structural model was estimated with full information maximum likelihood on all 318 cases. The indirect association was tested with bias-corrected bootstrapping using 1,000 resamples, each of which was re-estimated by full information maximum likelihood so that the point estimate and its interval rest on the same estimation basis and the same sample. Results appear in Figure 2 and Table 7.

**TABLE 7:** Structural model estimates (*N* = 318).

Path	β	SE	Bias-corrected 95% CI	Hypothesis
Total: PI → SRCP	0.694	0.038	[0.616, 0.763]	H1 supported
PI → WE (a)	0.714	0.034	[0.636, 0.770]	H2 supported
WE → SRCP (b)	0.321	0.076	[0.175, 0.464]	H3 supported
PI → SRCP, direct (c′)	0.465	0.074	[0.318, 0.606]	–
Indirect: PI → WE → SRCP	0.229	0.055	[0.128, 0.339]	H4 supported

Standardized coefficients. Point estimates and confidence intervals derive from the same estimation basis: full information maximum likelihood on all 318 cases, with bias-corrected bootstrapping (1,000 resamples) in which each resample was re-estimated by FIML. R^2^ = 0.509, 95% CI [0.405, 0.593], for work engagement and 0.532, 95% CI [0.425, 0.621], for self-rated caregiving performance. Unstandardized estimates appear in Supplementary Table 7.

Estimates from every model reported in this article, with the estimator, sample, standardization, and explained variance for each, are consolidated in Supplementary Table 10 so that coefficients from different specifications can be compared without ambiguity. A comparison of the present estimates with those reported in the original submission, together with an account of how the earlier discrepancy arose and how the revised analysis was verified, appears in Supplementary Note 4 and Supplementary Table 11.

All four hypotheses were supported. Professional identity accounted for just over half the variance in work engagement (R^2^ = 0.509), and the two predictors jointly accounted for 0.532 of the variance in self-rated caregiving performance. The indirect association through work engagement (β = 0.229) represented approximately 33% of the total association (β = 0.694), with a substantial direct association remaining (β = 0.465).

The cluster-adjusted interval for the indirect association reported in section “4.5 Clustering, practice status, and covariates,” [0.120, 0.353], likewise excludes zero, so the finding does not depend on ignoring the sampling structure.

Following current guidance, these results are described as a significant indirect association accompanied by a substantial remaining direct association, rather than being classified as partial mediation. The partial-complete distinction rests on significance tests of the direct path and conveys little about magnitude, and the term mediation implies a temporal process that a single-occasion design cannot evaluate.

## 5 Discussion

This study examined how professional identity relates to self-rated caregiving performance among work-integrated childcare trainees and early-career practitioners, and whether work engagement accounts for part of that relationship. Professional identity showed a strong total association with self-rated caregiving performance, a significant indirect association through work engagement, and a substantial remaining direct association. Because all measures were self-reported at a single occasion, these findings describe a covariance structure compatible with the proposed ordering; they do not establish that the ordering occurs in time.

### 5.1 Magnitude and comparison with prior evidence

Effect sizes warrant attention alongside significance, since the sample and correlations are large enough that significance alone is uninformative. The total association of β = 0.694 is substantially larger than typical meta-analytic estimates for engagement–performance relationships, which cluster around ρ = 0.30 to 0.45 with self-rated criteria (Christian et al., 2011). Two considerations bear on this. First, both variables here are self-appraisals collected in one instrument, which inflates observed associations relative to designs using independent criteria. Second, latent-variable estimation corrects for measurement error and therefore yields larger coefficients than the observed-score correlations usually reported in meta-analyses; the composite-score correlation was 0.601, closer to the published range.

The incremental contribution of work engagement is more modest than its statistical significance suggests. The indirect association accounts for roughly a third of the total, and the direct association remains the larger component. Work engagement thus appears to be one route among several. Professional identity may also relate to self-appraised performance through mechanisms not modeled here, including feedback-seeking, compliance with safety routines, receptivity to mentoring, and the tendency to interpret difficult tasks as professional development rather than as burden.

It is notable that the identity–engagement association (β = 0.714) was as strong in this boundary population as in studies of established employees, despite the provisional occupational membership and limited task autonomy that characterize placement work. Social identity theory would predict attenuation under provisional membership. That the association was not attenuated suggests that anticipated rather than achieved membership may function as a psychological resource, a possibility that longitudinal work could test directly.

### 5.2 Alternative explanations

The proposed ordering is one of several accounts consistent with these data, and the alternatives are theoretically serious rather than pro forma.

Reverse and reciprocal ordering are plausible. Trainees who believe they perform caregiving tasks competently may develop stronger occupational identification as a consequence, and satisfying practice experience may generate both engagement and identity simultaneously. Longitudinal evidence in teacher populations suggests that identity and engagement influence one another reciprocally over time, so the single-occasion estimates here may reflect an equilibrium of bidirectional processes rather than a one-directional flow.

Shared self-evaluative disposition is a second account. Professional identity and self-rated caregiving performance both consist of positively framed self-referential statements. Respondents with generally favorable self-perceptions, higher occupational self-efficacy, or a tendency toward socially desirable responding may rate themselves highly on both. The model comparisons in Table 4 constrain this account, since a merged factor fitted markedly worse, but they do not eliminate it: two constructs can be empirically separable and still share a common evaluative disposition. Item SRCP6, which concerns confidence, is the clearest point at which this overlap may operate.

Unmeasured common causes are a third account. Placement quality, supervisory support, organizational climate, and prior childcare experience could plausibly raise identity, engagement, and self-appraised performance together. None was measured. Distinguishing these accounts requires temporally separated measurement, independent performance criteria, or designs that manipulate placement conditions.

### 5.3 Theoretical implications

The contribution is best characterized as a boundary test rather than a new theory. The identity–engagement–performance sequence has been examined in teachers and other established workforces; what this study adds is evidence that the sequence holds in a population where the theoretical preconditions are weaker, namely provisional occupational membership and limited autonomy. That the associations were not attenuated indicates that the pathway does not depend on settled membership, which is informative for theories treating identification as a resource contingent on secure group belonging.

The study also illustrates a measurement point of general relevance. Constructs labeled as quality are frequently operationalised through practitioner self-report, which conflates an attribute of the caregiving process with an attribute of the practitioner’s self-appraisal. Keeping these distinct, as attempted here, changes what can be claimed: the present findings speak to how practitioners construe their own caregiving work, not to what children experience.

### 5.4 Practical implications

The implications below follow from correlational evidence and are candidates for intervention research rather than practices shown here to improve anything. No intervention was manipulated or evaluated in this study.

Since professional identity was associated with both engagement and favorable self-appraisal, its deliberate cultivation during work-integrated training merits testing. Structured orientation, mentoring relationships, guided reflection on the social value of caregiving, and exposure to experienced practitioners are plausible components. Similarly, because engagement accounted for part of the identity–performance association, placement design features that plausibly support vigor, dedication, and absorption — workload calibrated to trainee capacity, regular supervisory feedback, and meaningful task allocation rather than peripheral assignment — are worth evaluating.

One caution deserves emphasis. Because the outcome is self-appraised, interventions that raise identity and engagement may raise self-rated performance without altering observed caregiving quality. Evaluations should therefore include observational or supervisor-rated criteria rather than relying on practitioner self-report.

Regarding the provincial reform context, the present findings are consistent with the general proposition that vocational training models attending to identity formation and engagement may support workforce development. However, exposure to the post–course–competition–certificate–innovation model was not measured, and no claim about its effectiveness can be supported here. Likewise, the older adults and young children agenda and digitally supported care are directions for future workforce research, not implications of the present model.

### 5.5 Limitations

The design is cross-sectional, so temporal order cannot be established and the results are reported as indirect associations rather than mediation. All constructs were self-reported by the same individuals in a single instrument, so the design is susceptible to common-method variance; the statistical diagnostics reported cannot rule it out, and neither the design nor the diagnostics establish the magnitude of any inflation.

The outcome is a self-appraisal of caregiving performance and is not equivalent to observed process quality, inspection results, or child outcomes. Average variance extracted fell below 0.50 for all three constructs, and the Fornell–Larcker criterion was not satisfied for the two pairs involving professional identity, although it was satisfied, narrowly, for work engagement and self-rated caregiving performance. The constructs are separable by model comparison and heterotrait–monotrait evidence but are measured with considerable shared and indicator-specific variance.

Two instruments were substantially adapted and one was newly developed. Validation evidence for the self-rated caregiving performance scale, although it includes expert content review and pilot testing for comprehensibility, remains initial: no criterion validity was established against an independent performance indicator, and the scale has not been used outside this sample. One item is conceptually adjacent to self-efficacy.

Each construct was modeled as a single factor although its three dimensions proved empirically identifiable, so the primary model understates the structure present in the instruments; a second-order specification yielded slightly larger estimates and identical conclusions. Inference at the college level rests on only 11 clusters, so the cluster-robust standard errors and the cluster bootstrap are small-sample approximations. The sample is heterogeneous in practice status, and although group comparisons showed no significant differences, these were failures to detect differences rather than evidence of equivalence, and the employed subgroup was small. Placement duration, weekly hours, and prior experience were not recorded, so exposure could not be examined. Participants were nested within colleges and institutions; clustering was slight and robustness checks were conducted, but the design was not a probability sample. Recruitment through institutional channels may have produced selection toward more engaged respondents, and the number invited is unknown, so a response rate cannot be computed. The sample was 86.8% female, and generalization to male practitioners is limited. No *a priori* power analysis was conducted; only a post hoc sensitivity analysis is available. Finally, the sample is drawn from one Chinese province, and generalization to other national systems, which differ in qualification structures and regulatory arrangements, requires direct examination.

### 5.6 Future research

Longitudinal designs with temporally separated measurement are the clearest next step, permitting examination of whether identity precedes engagement and whether the two develop reciprocally. Multi-source designs incorporating supervisor ratings, structured observation using established instruments, and administrative safety records would separate self-appraisal from independently assessed performance. Studies measuring placement duration, intensity, and quality would clarify how workplace exposure shapes these constructs. Finally, the self-rated caregiving performance scale requires further validation, including tests of criterion validity against observed process quality and examination of its discriminant relation to occupational self-efficacy.

## 6 Conclusion

Among 318 work-integrated childcare trainees and early-career practitioners in Guangdong Province, professional identity was strongly associated with self-rated caregiving performance, and work engagement accounted for a significant portion of that association while a substantial direct association remained. These relationships were robust to a latent-variable covariate model, college fixed effects, and cluster-robust standard errors, and the three constructs were empirically distinguishable from merged alternatives.

The findings indicate that professional identity and work engagement are important correlates of how early-career childcare workers appraise their own caregiving practice during the transition from vocational preparation into employment. Because the design is cross-sectional and single-source, the results do not establish that identity or engagement causes better performance, and because the outcome is self-appraised, they do not speak to observed caregiving quality or to children’s experiences. Organizational practices such as structured mentoring, guided reflection, supervisory feedback, and considered placement design are promising candidates for intervention research on childcare workforce development, and their evaluation will require independent performance criteria rather than practitioner self-report alone.

## Data Availability

The datasets presented in this article are not readily available because the complete Chinese and English questionnaire items and the analysis code are provided in the Supplementary material. The individual-level dataset is not publicly available. Participants were informed at the point of consent that their data would be used in aggregated form, so public release of item-level responses would exceed the scope of the consent obtained. De-identified data may be made available by the corresponding author on reasonable request for verification or replication, subject to approval by the Scientific Research Ethics Committee of South China Normal University and to a data-use agreement. The same conditions govern access during peer review: the de-identified dataset, the analysis code, and the software output can be transmitted to reviewers or to an appointed statistical reviewer through the editorial office, under the confidentiality terms of the review process and with the ethics committee notified, and the authors have prepared these materials for that purpose. Requests to access the datasets should be directed to HY, heyuda1998@163.com.
