# Supplementary material for: Professional identity and self-rated caregiving performance among work-integrated childcare trainees and early-career practitioners: an indirect association through work engagement
Source: Front Psychol. 2026 Oct 27;17:1919887. doi: 10.3389/fpsyg.2026.1919887 (PMC13747828; doi:10.3389/fpsyg.2026.1919887)
Supplement: Supplementary file 1 [file Table_1.docx]

**Supplementary Material**

**Professional Identity and Self-Rated Caregiving Performance among Work-Integrated Childcare Trainees and Early-Career Practitioners: An Indirect Association through Work Engagement**

Chen Chunhua¹ and He Yuda²*

# **Note on Instrument Language and Translation**

All instruments were administered in Chinese. The Chinese items below are the versions completed by participants. English renderings were prepared after data collection for reporting only and were never administered; they therefore provide no validation evidence for the Chinese instruments.

Content validity was established before data collection. Seven reviewers (four early childhood education scholars, two childcare centre directors, and one organisational psychologist) examined the draft items of all three instruments online for content relevance, clarity, and freedom from ambiguity. The revised instrument set was piloted with 25 childcare trainees from the same programme population who were not included in the main sample. Six items were subsequently reworded for clarity — PI2, PI3, WE2, WE5, SRCP8, and SRCP9, marked with a superscript a in the tables below; these changes affected wording only, leaving content, dimensional assignment, and scoring direction unchanged. Pilot responses were used solely to assess comprehensibility and appear in no analysis reported here. Authors preparing replications should use the Chinese items, not the English renderings.

Response scale for all items: 1 = strongly disagree, 2 = disagree, 3 = uncertain, 4 = agree, 5 = strongly agree. No items were reverse-scored. Participants rated their actual experience during recent childcare practice (internship, employment-linked placement, or employment).

# **Supplementary Table S1a. Professional identity items and CFA results**

| **Code** | **Chinese item (as administered)** | **English rendering** | **Dimension** | **λ (unstd.)** | **SE** | **λ (std.)** | **Res. var.** |
| --- | --- | --- | --- | --- | --- | --- | --- |
| PI1 | 我认为托育工作对社会具有重要的价值。 | I believe that childcare work has important social value. | Value identification | 0.541 | 0.044 | 0.624 | 0.611 |
| PI2 ᵃ | 我认为托育工作是有意义且不可或缺的。 | I view childcare work as meaningful and indispensable. | Value identification | 0.797 | 0.056 | 0.701 | 0.509 |
| PI3 ᵃ | 我相信托育工作能为儿童及其家庭的福祉带来积极影响。 | I believe that childcare work contributes to the wellbeing of children and their families. | Value identification | 0.615 | 0.049 | 0.641 | 0.589 |
| PI4 | 我对托育工作有一种归属感。 | I feel a sense of belonging to childcare work. | Occupational belonging | 0.770 | 0.052 | 0.712 | 0.493 |
| PI5 | 我觉得自己与托育从业人员这个群体紧密相连。 | I feel closely connected with childcare practitioners as an occupational group. | Occupational belonging | 0.747 | 0.060 | 0.638 | 0.593 |
| PI6 | 我乐于向他人表明自己是托育工作者。 | I feel comfortable identifying myself as a childcare practitioner. | Occupational belonging | 0.770 | 0.054 | 0.700 | 0.510 |
| PI7 | 我打算在托育相关领域继续发展。 | I intend to continue developing in childcare-related work. | Career commitment | 0.814 | 0.054 | 0.720 | 0.482 |
| PI8 | 我愿意不断提升自己的专业照护能力。 | I am willing to keep improving my professional caregiving competence. | Career commitment | 0.768 | 0.057 | 0.676 | 0.543 |
| PI9 | 我会把托育工作作为长期的职业方向。 | I regard childcare work as a long-term career direction. | Career commitment | 0.673 | 0.057 | 0.613 | 0.624 |

*Note. All loadings significant at p < .001. The three-dimensional framework follows Wei et al. (2013); item wording was informed by Zeng and Liu (2024) and other recent professional-identity research. Teaching referents were replaced with childcare and caregiving referents; item count and scoring direction were unchanged. Estimation by FIML, N = 318. Standard errors from the numerically approximated observed information matrix. ᵃ Item reworded for clarity following expert review and pilot testing; wording only, with content, dimension, and scoring direction unchanged.*

# **Supplementary Table S1b. Work engagement items and CFA results**

| **Code** | **Chinese item (as administered)** | **English rendering** | **Dimension** | **λ (unstd.)** | **SE** | **λ (std.)** | **Res. var.** |
| --- | --- | --- | --- | --- | --- | --- | --- |
| WE1 | 在照护实践中，我感到精力充沛。 | I feel energetic during childcare practice. | Vigor | 0.575 | 0.042 | 0.677 | 0.542 |
| WE2 ᵃ | 我有动力完成每天的照护常规工作。 | I feel motivated to complete daily childcare routines. | Vigor | 0.680 | 0.053 | 0.638 | 0.593 |
| WE3 | 即使照护任务繁重，我也能保持努力。 | I maintain effort even when childcare tasks are demanding. | Vigor | 0.764 | 0.053 | 0.700 | 0.510 |
| WE4 | 面对实践岗位上的工作，我能够坚持不懈。 | I show persistence in my work at the practice site. | Vigor | 0.758 | 0.062 | 0.622 | 0.613 |
| WE5 ᵃ | 我为参与照护实践感到自豪。 | I feel proud of participating in childcare practice. | Dedication | 0.708 | 0.047 | 0.715 | 0.489 |
| WE6 | 我觉得照护任务是有意义的。 | I find caregiving tasks meaningful. | Dedication | 0.875 | 0.060 | 0.709 | 0.497 |
| WE7 | 我认真对待照顾儿童的责任。 | I take my responsibilities for children seriously. | Dedication | 0.724 | 0.048 | 0.723 | 0.477 |
| WE8 | 我全身心投入儿童发展与安全保障工作。 | I devote myself to children's development and safety support. | Dedication | 0.814 | 0.051 | 0.749 | 0.439 |
| WE9 | 执行照护常规工作时，我能保持专注。 | I concentrate while carrying out childcare routines. | Absorption | 0.607 | 0.049 | 0.633 | 0.600 |
| WE10 | 我会沉浸于观察儿童并与他们互动。 | I become absorbed in observing and interacting with children. | Absorption | 0.777 | 0.056 | 0.680 | 0.538 |
| WE11 | 我能专注于与家长沟通及照护计划工作。 | I stay focused on communication with families and care planning. | Absorption | 0.669 | 0.046 | 0.708 | 0.499 |
| WE12 | 我会密切关注与安全有关的实践细节。 | I pay close attention to safety-related details in practice. | Absorption | 0.832 | 0.055 | 0.724 | 0.476 |

*Note. All loadings significant at p < .001. Framework from the Utrecht Work Engagement Scale (Schaufeli et al., 2002, 2006) and its Chinese applications. For each dimension, the four statements judged most compatible with childcare practice were selected and adapted: generic work referents became childcare-practice referents and client referents became children and families. Authors intending to reuse the UWES should consult the current licensing terms of the scale owners. ᵃ Item reworded for clarity following expert review and pilot testing; wording only, with content, dimension, and scoring direction unchanged.*

# **Supplementary Table S1c. Self-rated caregiving performance items and CFA results**

| **Code** | **Chinese item (as administered)** | **English rendering** | **Dimension** | **λ (unstd.)** | **SE** | **λ (std.)** | **Res. var.** |
| --- | --- | --- | --- | --- | --- | --- | --- |
| SRCP1 | 我能把所学的托育知识运用到实践中。 | I can apply the childcare knowledge I have learned in practice. | Knowledge application | 0.553 | 0.046 | 0.623 | 0.611 |
| SRCP2 | 我能运用专业知识理解儿童的需要。 | I can use professional knowledge to understand children's needs. | Knowledge application | 0.664 | 0.057 | 0.607 | 0.632 |
| SRCP3 | 我能把培训内容与日常照护任务结合起来。 | I can connect training content with daily care tasks. | Knowledge application | 0.654 | 0.053 | 0.628 | 0.606 |
| SRCP4 | 我能恰当地完成日常照护任务。 | I can perform routine caregiving tasks appropriately. | Caregiving skills | 0.751 | 0.047 | 0.754 | 0.431 |
| SRCP5 | 我能与儿童及其照护者进行有效沟通。 | I can communicate effectively with children and their caregivers. | Caregiving skills | 0.697 | 0.053 | 0.661 | 0.563 |
| SRCP6 | 我能有信心地完成各项实践照护任务。 | I can complete practical care tasks with confidence. | Caregiving skills | 0.570 | 0.050 | 0.599 | 0.642 |
| SRCP7 | 我能在日常活动中支持儿童的发展。 | I can support children's development in daily activities. | Development and safety | 0.641 | 0.052 | 0.632 | 0.601 |
| SRCP8 ᵃ | 我能认真执行健康与安全常规。 | I attend carefully to health and safety routines. | Development and safety | 0.668 | 0.046 | 0.711 | 0.494 |
| SRCP9 ᵃ | 我能识别并应对基本的照护风险。 | I can identify and respond to basic care risks. | Development and safety | 0.665 | 0.049 | 0.677 | 0.541 |

*Note. All loadings significant at p < .001. Items were written for this study, drawing on the three quality components of Donabedian (1988) and the responsive caregiving, safety, and early learning components of the nurturing care framework (WHO, UNICEF, and World Bank Group, 2018), and phrased as self-appraisals of the practitioner’s own task enactment. Content validity was reviewed by the seven-member expert panel described above, and the items were piloted for comprehensibility, before the final version was fixed. SRCP6 concerns confidence and is conceptually adjacent to self-efficacy; it was retained because its removal did not materially alter the factor structure. Item codes SRCP1–SRCP9 correspond to codes SRCQ1–SRCQ9 in the raw data file and in the original questionnaire. ᵃ Item reworded for clarity following expert review and pilot testing; wording only, with content, dimension, and scoring direction unchanged.*

# **Supplementary Table S2. Recruitment flow and data screening**

| **Stage** | **n** | **Note** |
| --- | --- | --- |
| Questionnaires returned | 352 | Collected 2 March – 29 April 2026 |
| Excluded: invariant responding | 13 | Identical option across all 30 items |
| Excluded: missingness > 30% | 10 | More than 9 of 30 items unanswered |
| Excluded: completion time < 150 s | 9 | Below pre-set minimum plausible duration |
| Excluded: duplicate submission | 2 | Matched on submission metadata |
| Analytic sample | 318 | Retention 90.3% of returned questionnaires |

*Note. The questionnaire was distributed through open placement communication channels, so the number of individuals who saw the invitation is unknown and a conventional response rate cannot be computed. Remaining item-level missingness was 79 of 9,540 responses (0.83%), affecting 56 participants across 32 patterns; full information maximum likelihood was used.*

# **Supplementary Table S3. Distributional diagnostics**

| **Construct** | **Skewness range** | **Excess kurtosis range** |
| --- | --- | --- |
| Professional identity | −.60 to .14 | −.77 to .19 |
| Work engagement | −.86 to .07 | −.72 to .55 |
| Self-rated caregiving performance | −.74 to .11 | −.63 to .40 |
| All 30 items | −.86 to .14 | −.77 to .55 |

*Note. Values within conventional limits (|skewness| < 2, |kurtosis| < 7) at which maximum likelihood performs adequately with five-point items. No post hoc model modifications were made: no residual covariances were freed and no items were removed after inspecting fit.*

# **Supplementary Table S4. Alternative measurement models**

| **Model** | **χ²** | **df** | **CFI** | **TLI** | **RMSEA** | **SRMR** | **Δχ²** | **Δdf** | **p** | **ΔCFI** |
| --- | --- | --- | --- | --- | --- | --- | --- | --- | --- | --- |
| Three-factor (hypothesised) | 595.04 | 402 | .954 | .950 | .039 | .040 | — | — | — | — |
| Two-factor (PI + SRCP) | 881.11 | 404 | .886 | .877 | .061 | .056 | 286.07 | 2 | <.001 | −.068 |
| Two-factor (PI + WE) | 943.50 | 404 | .871 | .861 | .065 | .060 | 348.45 | 2 | <.001 | −.083 |
| Two-factor (WE + SRCP) | 1010.88 | 404 | .855 | .844 | .069 | .066 | 415.84 | 2 | <.001 | −.099 |
| One-factor | 1286.10 | 405 | .790 | .774 | .083 | .075 | 691.06 | 3 | <.001 | −.164 |

# **Supplementary Table S5. Discriminant validity and common-method diagnostics**

| **Pair** | **Latent r** | **√AVE 1** | **√AVE 2** | **Fornell–Larcker** | **HTMT** | **HTMT 95% CI** |
| --- | --- | --- | --- | --- | --- | --- |
| PI – WE | .714 | .671 | .691 | Not satisfied | .712 | [.643, .774] |
| PI – SRCP | .694 | .671 | .656 | Not satisfied | .687 | [.607, .759] |
| WE – SRCP | .653 | .691 | .656 | Satisfied | .645 | [.560, .719] |

*Note. HTMT confidence intervals from 5,000 bootstrap resamples; all upper bounds below .85. AVE values were .450 (PI), .477 (WE), and .431 (SRCP), all below .50; composite reliabilities were .880, .916, and .871. Fornell and Larcker (1981) note that convergent validity may be regarded as adequate when AVE is below .50 but composite reliability exceeds .60. Common latent factor: standardised loading changes ranged from .006 to .043 (M = .019). Full-collinearity VIFs: 2.41 (PI), 2.28 (WE), 2.19 (SRCP). These diagnostics constrain but cannot eliminate common-method explanations.*

# **Supplementary Table S6. Clustering and robustness analyses**

Panel A. Latent-variable models (FIML, N = 318). Coefficients are directly comparable with the primary model in Table 7.

| **Model** | **PI → WE** | **WE → SRCP** | **PI → SRCP** | **Indirect** |
| --- | --- | --- | --- | --- |
| Primary model (no covariates) | .714 | .321 | .465 | .229 |
| MIMIC with 12 covariates | .728 | .302 | .471 | .220 |

Panel B. Observed-variable checks on composite scores. Coefficients in this metric are systematically smaller than the latent estimates because composite scores are not corrected for measurement error; the informative comparison is between rows within this panel, not between panels.

| **Analysis** | **PI → SRCP** | **WE → SRCP** | **PI → WE** |
| --- | --- | --- | --- |
| Baseline (composite scores) | .395 (SE .056) | .323 (SE .056) | .639 (SE .043) |
| Cluster-robust SE by college | .395 (SE .060) | .323 (SE .070) | .639 (SE .049) |
| College fixed effects | .397 (SE .056) | .299 (SE .057) | — |
| With background covariates | .388 (SE .058) | .308 (SE .058) | .647 (SE .044) |

*Note. Covariates in both panels: gender, age band, certificate status, major (three indicators), practice status (two indicators), and city (four indicators), twelve in total. The MIMIC model regresses the three latent variables on these covariates and was estimated by FIML on all 318 cases. All coefficients p < .001. College ICCs were .000 (PI), .015 (WE), and .034 (SRCP), giving design effects of 1.00, 1.41, and 1.96. Institution ICCs were .000, .008, and .009 (design effects 1.00, 1.04, 1.05). Practice-status group comparisons: PI, F(2, 315) = 0.47, p = .627, η² = .003; WE, F(2, 315) = 1.05, p = .350, η² = .007; SRCP, F(2, 315) = 0.44, p = .643, η² = .003.*

# **Supplementary Table S7. Structural model, standardised and unstandardised**

| **Path** | **β (std.)** | **B (unstd.)** | **SE (std.)** | **Bias-corrected 95% CI (std.)** |
| --- | --- | --- | --- | --- |
| Total: PI → SRCP | .694 | .612 | .038 | [.616, .763] |
| PI → WE (a) | .714 | .848 | .034 | [.636, .770] |
| WE → SRCP (b) | .321 | .269 | .076 | [.175, .464] |
| PI → SRCP direct (c′) | .465 | .384 | .074 | [.318, .606] |
| Indirect: PI → WE → SRCP | .229 | .228 | .055 | [.128, .339] |
| R² work engagement | .509 | — | .048 | [.405, .593] |
| R² self-rated caregiving performance | .532 | — | .050 | [.425, .621] |

*Note. All estimates derive from full information maximum likelihood on all 318 cases. Bias-corrected bootstrap intervals are based on 1,000 resamples, each re-estimated by FIML, so that point estimates and intervals share the same estimation basis and the same sample. Internal consistency check: c′ + a×b = .465 + .229 = .694, equal to the total association, as required in a saturated three-variable structure.*

# **Supplementary Table S8. Monte Carlo simulation: estimation accuracy and precision**

Population values were set to the parameter estimates of the fitted three-factor model; complete data were generated from the implied mean vector and covariance matrix and the model re-estimated in each replication.

| **N** | **Reps** | **a (pop .714)** | **b (pop .321)** | **c′ (pop .465)** | **Indirect (pop .229)** | **SD of indirect** | **Power** |
| --- | --- | --- | --- | --- | --- | --- | --- |
| 150 | 500 | .711 (−0.4%) | .318 (−1.0%) | .470 (+1.1%) | .226 (−1.5%) | .080 | .814 |
| 200 | 500 | .715 (+0.2%) | .317 (−1.4%) | .469 (+0.8%) | .226 (−1.2%) | .071 | .894 |
| 250 | 500 | .713 (−0.1%) | .321 (−0.1%) | .464 (−0.3%) | .228 (−0.3%) | .061 | .978 |
| 318 | 1000 | .713 (−0.0%) | .324 (+0.9%) | .462 (−0.7%) | .231 (+0.8%) | .055 | .990 |
| 400 | 500 | .712 (−0.2%) | .326 (+1.5%) | .460 (−1.2%) | .232 (+1.3%) | .048 | 1.000 |

*Note. Values are means across replications with relative bias in parentheses. Convergence was 100% at every sample size. At N = 318 the empirical standard deviation of the indirect association (.0548) matches the bias-corrected bootstrap standard error obtained from the observed data (.055); the two are derived independently. Power is the proportion of replications in which the estimate exceeded 1.96 empirical standard deviations from zero. Because population values were taken from the fitted model, the simulation establishes accurate and precise estimation at this sample size rather than sufficiency to detect a substantially smaller association.*

# **Supplementary Table S9. Dimensional structure of the three instruments**

| **Model** | **χ²** | **df** | **CFI** | **TLI** | **RMSEA** | **Comparison** |
| --- | --- | --- | --- | --- | --- | --- |
| Three-factor (primary model) | 595.04 | 402 | .954 | .950 | .039 | — |
| Nine correlated first-order dimensions | 424.92 | 369 | .987 | .984 | .022 | Δχ²(33) = 170.12, p < .001 |
| Second-order (three dimensions per construct) | 444.77 | 384 | .986 | .984 | .022 | Δχ²(18) = 150.28, p < .001 |
| Second-order vs nine-factor | — | — | — | — | — | Δχ²(15) = 19.84, p = .178 |

*Note. Within-construct tests of one dimension against three correlated dimensions: professional identity Δχ²(3) = 44.18; work engagement Δχ²(3) = 36.60; self-rated caregiving performance Δχ²(3) = 64.63, all p < .001. The second-order restriction was not rejected, so the three second-order constructs are supported. Structural paths estimated from the second-order model: a = .746, b = .323, c′ = .492, indirect = .241, total = .733; R² = .556 and .584. These are larger than the first-order estimates reported in Table 7, which are retained as the more conservative values.*

# **Supplementary Table S10. Consolidated structural estimates across models**

| **Model** | **Estimator, sample** | **a** | **b** | **c′** | **Indirect** | **Total** | **R² (WE)** |
| --- | --- | --- | --- | --- | --- | --- | --- |
| Latent SEM (primary, Table 7) | FIML, N = 318 | .714 | .321 | .465 | .229 | .694 | .509 |
| Latent SEM, cluster bootstrap | FIML, N = 318, G = 11 | .714 | .321 | .465 | .229 | .694 | .509 |
| Latent MIMIC, 12 covariates | FIML, N = 318 | .728 | .302 | .471 | .220 | .691 | n.r. |
| Latent second-order (not adopted) | FIML, N = 318 | .746 | .323 | .492 | .241 | .733 | .556 |
| Composite-score path model, baseline | OLS, n = 318 | .639 | .323 | .395 | .206 | .601 | .408 |
| Composite-score path model, 12 covariates | OLS, n = 318 | .647 | .308 | .388 | .199 | .587 | .444 |

*Note. All coefficients are standardised. Each row is internally consistent: within every model a × b equals the reported indirect association and c′ + a × b equals the reported total. R² for work engagement equals a² only in the models without covariates, because in the covariate models the twelve covariates also predict work engagement; in the composite-score baseline a² = .4083 equals R² to four decimal places, whereas in the composite-score covariate model a² = .419 and R² = .444, the difference being the variance explained by the covariates. Coefficients are not comparable in magnitude across the latent and composite-score blocks because composite scores are not corrected for measurement error. A previous response letter reported .647 and .323 in a single sentence without stating that the first comes from the composite-score covariate model and the second from the composite-score baseline model; their product is not an estimate of any quantity, and the pairing was a reporting error corrected here. Confidence intervals: ordinary bootstrap (1,000 FIML resamples) indirect [.128, .339], total [.616, .763]; college-level cluster bootstrap (1,000 resamples) indirect [.120, .353], total [.607, .747].*

# **Supplementary Table S11. Audit of original and revised principal results**

The original submission reported analyses computed from an intermediate version of the analytic dataset. The values below compare what was reported then with what the raw data yield. The raw item responses are unchanged; only the analytic dataset and the analysis were rebuilt.

| **Quantity** | **Original submission** | **Revised (from raw data)** | **Substantive conclusion** |
| --- | --- | --- | --- |
| χ², df | not reported | 595.04, 402 | — |
| χ²/df | 2.14 | 1.48 | Fit improves |
| CFI / TLI | .942 / .935 | .954 / .950 | Fit improves |
| RMSEA / SRMR | .060 / .045 | .039 / .040 | Fit improves |
| α (PI / WE / SRCP) | .835 / .888 / .852 | .879 / .914 / .873 | Reliability higher |
| CR (PI / WE / SRCP) | .901 / .929 / .902 | .880 / .916 / .871 | Marginally lower, all ≥ .87 |
| AVE (PI / WE / SRCP) | .502 / .522 / .505 | .450 / .477 / .431 | All now below .50; reported openly |
| Loading range PI / WE / SRCP | .688–.741 / .696–.755 / .688–.733 | .613–.720 / .622–.749 / .599–.754 | Lower but all significant |
| Latent r: PI–WE / PI–SRCP / WE–SRCP | .700 / .721 / .631 | .714 / .694 / .653 | Pattern unchanged |
| a (PI → WE) | .700 | .714 | Unchanged in sign and significance |
| b (WE → SRCP) | .347 | .321 | Unchanged in sign and significance |
| c′ (direct) | .478 | .465 | Unchanged in sign and significance |
| Indirect association | .243, 95% CI [.185, .312] | .229, 95% CI [.128, .339] | H4 supported in both |
| Total association | .721 | .694 | Unchanged |
| R² (WE / SRCP) | not reported | .509 / .532 | Newly reported |
| Internal consistency of estimates | c′ + a×b = .721 but b + a×c′ implies r(WE, SRCP) = .682 against .631 reported | c′ + a×b = .465 + .229 = .694 = total; b + a×c′ = .321 + .332 = .653 = latent r(WE, SRCP) | Original set was not internally consistent; revised set is |

*Note. All four hypotheses were supported before and after the correction, and no hypothesis lost significance. Two results became less favourable: average variance extracted now falls below .50 for all three constructs, and the Fornell–Larcker criterion is not satisfied for the two pairs involving professional identity. One earlier statement was too unfavourable and has been corrected: the criterion is satisfied for work engagement and self-rated caregiving performance. Verification of the revised analysis is described in Supplementary Note S4.*

# **Supplementary Note S4. Verification of the revised analysis**

The raw item-level responses were never altered. The discrepancy arose in the data-management and analysis workflow: the original analysis inadvertently used an intermediate export of the analytic dataset, produced before the final cleaning procedures were complete, so that duplicate and ineligible cases had not yet been removed and variable coding and missing-value rules had not yet been harmonised.

The following checks were carried out on the rebuilt dataset and analysis. First, integrity of the data: all item values fall within the 1 to 5 response range with no out-of-range entries; there are no duplicate cases; the archived export of 352 returned questionnaires partitions exactly into the 318 retained and the 34 excluded, with no overlap and none omitted; and a cell-by-cell comparison of the 318 retained cases against the archived export returned zero differences. Second, the exclusion rules were verified as applied: the shortest completion time in the analytic sample is 155 seconds, no case shows an invariant response pattern across all 30 items, and no case exceeds 30% missingness.

Third, the estimation was reproduced independently. The measurement model was fitted twice, once using an established structural equation modelling package and once using a maximum likelihood routine written for this purpose; the two solutions agreed to within .0001 on every standardised loading and reproduced identical latent correlations. Fourth, the missing-data treatment was checked by comparing full information maximum likelihood with listwise deletion, which produced latent correlations differing by no more than .02. Fifth, the precision of the indirect association was cross-checked by two independent routes: the bias-corrected bootstrap standard error obtained from the observed data (.055) and the empirical standard deviation of the indirect association across 1,000 Monte Carlo replications (.0548).

Finally, the reported estimates were checked for internal consistency. In the revised set, c′ + a × b reproduces the total association exactly and b + a × c′ reproduces the latent correlation between work engagement and self-rated caregiving performance to within rounding. The corresponding identities did not hold in the original set, which is what first indicated that the reported values could not all have come from one estimation.

# **Supplementary Note S0. Informed consent text**

Participants read the following before beginning (Chinese original; English rendering shown): the study concerns the occupational psychology and caregiving practice of people undertaking childcare placements or employment; the survey is anonymous and collects no personally identifiable information such as name or student number; participation is voluntary and may be discontinued at any time; data are used in aggregated form for academic research only; completing all items indicates consent for the data to be used in analysis and publication; personal information is not provided to the participant’s college, childcare institution, or any third party; the survey takes approximately 5–10 minutes; participation requires being 18 years of age or older.

Because consent specified use in aggregated form, item-level individual responses are not released publicly. See the Data Availability Statement in the main article.

Ethics approval: Scientific Research Ethics Committee of South China Normal University, Approval No. KY2025062302, issued 26 February 2025; approved research period 1 March 2025 to 30 December 2026. Both the pilot administration and the main data collection fall within this period.

# **Supplementary Note S1. Replication code**

The model can be reproduced in lavaan with the following specification. A complete standalone replication file, Analysis_Code.R, covering all reported analyses accompanies the submission. Equivalent AMOS and Mplus specifications and the de-identified dataset are not publicly available; they may be made available under the conditions stated in the Data Availability Statement, including transmission to reviewers through the editorial office.

```
library(lavaan)
model <- '
  PI   =~ PI1+PI2+PI3+PI4+PI5+PI6+PI7+PI8+PI9
  WE   =~ WE1+WE2+WE3+WE4+WE5+WE6+WE7+WE8+WE9+WE10+WE11+WE12
  SRCP =~ SRCP1+SRCP2+SRCP3+SRCP4+SRCP5+SRCP6+SRCP7+SRCP8+SRCP9
  WE   ~ a*PI
  SRCP ~ cp*PI + b*WE
  indirect := a*b
  total    := cp + a*b
'
fit <- sem(model, data = d, missing = "fiml", estimator = "ML",
           se = "bootstrap", bootstrap = 1000)
summary(fit, fit.measures = TRUE, standardized = TRUE, rsquare = TRUE)
parameterEstimates(fit, boot.ci.type = "bca.simple", standardized = TRUE)
```

# **Supplementary Note S2. Interpretive boundary of the outcome**

The outcome variable is a self-appraisal of caregiving performance and is not a measure of care quality. Care quality in early childhood settings is conventionally assessed by trained external observers using standardised instruments; self-appraisal cannot substitute for such observation, and self-ratings are subject to leniency and self-enhancement. The construct label used here — self-rated caregiving performance — was chosen to make this boundary explicit. No claim is made about observed process quality, institutional inspection outcomes, or children’s developmental outcomes.

The three constructs were empirically distinguishable, as the model comparisons in Table S4 show, but average variance extracted fell below .50 for all three, and the Fornell–Larcker criterion was not satisfied for the two pairs involving professional identity, although it was satisfied, narrowly, for work engagement and self-rated caregiving performance. The most defensible reading is that these are distinct constructs measured with imperfect precision through a shared self-report method, and estimates involving professional identity and self-rated caregiving performance should be read with that proximity in mind.

# **Supplementary Note S3. Contextual boundary**

The Guangdong vocational-education reform context, the post–course–competition–certificate–innovation training model, and the older adults and young children care agenda situate the study and inform its practical discussion. None was measured. Exposure to the training model, digital-care competence, intergenerational-care coordination, and institutional training-system characteristics were not included as variables, and no claim about the effectiveness of these initiatives is supported by the present model.
